# Supplementary material for: Influence of Fermentation Time on the Phenolic Compounds, Vitamin C, Color and Antioxidant Activity in the Winemaking Process of Blueberry (Vaccinium corymbosum) Wine Obtained by Maceration
Source: Molecules. 2022 Nov 10;27(22):7744. doi: 10.3390/molecules27227744 (PMC9696742; doi:10.3390/molecules27227744)
Supplement: Supplementary file 1 [file molecules-27-07744-s001.zip › molecules-2000091-supplementary -2nd revised.pdf]

Supplementary Material

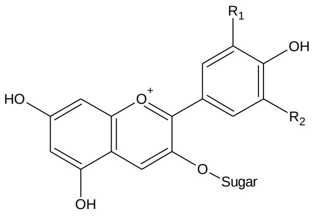

Anthocyanin	R1	R2
Cyanidin	OH	H
Delphinidin	OH	OH
Peonidin	OCH_3	H
Petunidin	OCH_3	OH
Malvidin	OCH_3	OCH_3

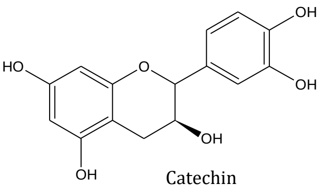

Catechin

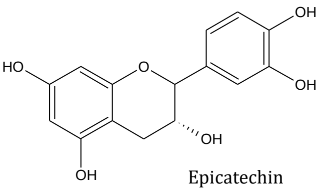

Epicatechin

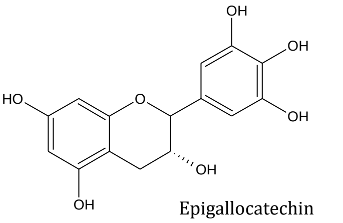

Epigallocatechin

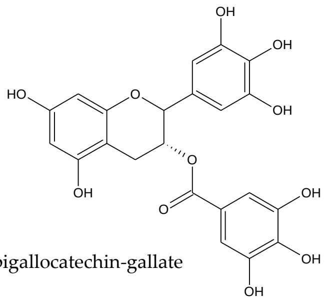

Epigallocatechin-gallate

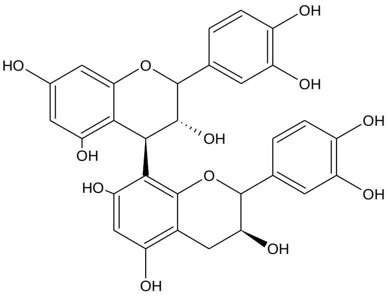

Procyanidin B1

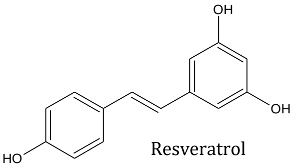

Resveratrol

Structures of Catechin, Epicatechin, Epigallocatechin, Epigallocatechin-gallate, Procyanidin B1 and Resveratrol.
